# Amogel: a multi-omics classification framework using associative graph neural networks with prior knowledge for biomarker identification

**DOI:** 10.1186/s12859-025-06111-6

**Published:** 2025-03-28

**Authors:** Chia Yan Tan, Huey Fang Ong, Chern Hong Lim, Mei Sze Tan, Ean Hin Ooi, KokSheik Wong

**Affiliations:** 1 School of Information Technology, Monash University Malaysia, Jalan Lagoon Selatan, 47500 Petaling Jaya, Selangor Malaysia; 2 School of Engineering, Monash University Malaysia, Jalan Lagoon Selatan, 47500 Petaling Jaya, Selangor Malaysia

**Keywords:** Graph neural network, Association rule mining, Graph classification, Multi-omics, Prior knowledge

## Abstract

The advent of high-throughput sequencing technologies, such as DNA microarray and DNA sequencing, has enabled effective analysis of cancer subtypes and targeted treatment. Furthermore, numerous studies have highlighted the capability of graph neural networks (GNN) to model complex biological systems and capture non-linear interactions in high-throughput data. GNN has proven to be useful in leveraging multiple types of omics data, including prior biological knowledge from various sources, such as transcriptomics, genomics, proteomics, and metabolomics, to improve cancer classification. However, current works do not fully utilize the non-linear learning potential of GNN and lack of the integration ability to analyse high-throughput multi-omics data simultaneously with prior biological knowledge. Nevertheless, relying on limited prior knowledge in generating gene graphs might lead to less accurate classification due to undiscovered significant gene-gene interactions, which may require expert intervention and can be time-consuming. Hence, this study proposes a graph classification model called associative multi-omics graph embedding learning (AMOGEL) to effectively integrate multi-omics datasets and prior knowledge through GNN coupled with association rule mining (ARM). AMOGEL employs an early fusion technique using ARM to mine intra-omics and inter-omics relationships, forming a multi-omics synthetic information graph before the model training. Moreover, AMOGEL introduces multi-dimensional edges, with multi-omics gene associations or edges as the main contributors and prior knowledge edges as auxiliary contributors. Additionally, it uses a gene ranking technique based on attention scores, considering the relationships between neighbouring genes. Several experiments were performed on BRCA and KIPAN cancer subtypes to demonstrate the integration of multi-omics datasets (miRNA, mRNA, and DNA methylation) with prior biological knowledge of protein-protein interactions, KEGG pathways and Gene Ontology. The experimental results showed that the AMOGEL outperformed the current state-of-the-art models in terms of classification accuracy, F1 score and AUC score. The findings of this study represent a crucial step forward in advancing the effective integration of multi-omics data and prior knowledge to improve cancer subtype classification.

## Introduction

Cancer remains one of the leading causes of death in this modern day, with millions of new cases diagnosed each year. Although significant advancement in early detection and treatment has been made, the heterogeneity of cancer poses a substantial challenge. Cancer researchers have identified numerous subtypes of cancer where their molecular and clinical characteristics are different for the same cancer type [1, 2]. Understanding these differences and accurately identifying these subtypes are paramount for developing an effective treatment. Over the years, high throughput technologies such as microarray, next-generation sequencing and mass spectrometry have enabled multiple omics (multi-omics) data generation and analysis for various molecular processes. For example, it allows the comparison of gene expression patterns between different groups, such as cancer subtypes [3, 4]. With the advent of these technologies, the focus of cancer research has thus shifted from single-omics analysis to multi-omics integrative analysis.

Existing studies [5–11] have demonstrated improved cancer subtype classification with multi-omics data integration. However, a carefully crafted solution is required due to the high dimensionality of omics data, where there is a large number of features compared with a relatively low sample size [12]. The high-dimensional nature of omics data often results in model overfitting, where the learning of the classification model is biased by noise rather than meaningful biological information [13]. Additionally, it also leads to computational inefficiencies and causes memory shortages due to the vast search spaces required for data analysis. Therefore, numerous studies have addressed these challenges by introducing novel dimensionality reduction techniques [14–18], which have proven not only to enhance cancer classification performance but also lead to better biomarker discovery by removing irrelevant features and identifying potential candidate biomarkers.

The recent development of deep learning models has led to techniques such as deep neural networks (DNN) and convolutional neural networks (CNN) being adopted for multi-omics integrative analysis. Compared to conventional machine learning models, deep learning models have a higher capability of learning complex patterns and representation with minimal feature engineering [19]. Often, most of the proposed deep learning models on multi-omics integrative analysis use early fusion methods with concatenation technique, where multi-omics data for each sample are combined and fed into the deep learning model to encode features for classification tasks. In contrast, late fusion methods employ separate deep learning models to encode feature representations for each omics data type, followed by a concatenation of feature representations and a final deep learning layer to learn the final classification. For instance, [10] proposed a DNN-based multi-omics integration framework with an auxiliary classifiers-enhanced autoencoder (MOCAT) for cancer subtype classification. In this framework, multi-omics data were fed into the DNN autoencoder to learn compact representation and extract omics-specific features. These encoded omics-specific features were fused by concatenation and feed into the final multi-head attention autoencoder. The multi-head attention mechanism emphasized the distinct significance of various omics modalities since different types of omics data contribute differently to the aggregated predictive accuracy.

Traditional deep learning models such as DNN are effective at extracting feature embedding in row-column format, while CNN are suitable for unstructured data, such as image or audio data. Both models operate in the Euclidean domain, but due to the complex nature of biological organisms, the relationships among genes are better represented in a graph structure than in the Euclidean domain. To address this gap, graph neural networks (GNN), a sub-field of deep learning model, are increasingly used in biomedical research. GNN provide improved performance and interpretability due to their ability to model graph structures. [5] proposed multi-omics graph convolutional networks (MOGONET) for biomedical classification. The study proposed modality-specific graph convolutional networks (GCN) for multi-omics mRNA, miRNA and DNA methylation data for feature learning with a view correlation discovery network as the late fusion technique for final prediction. On the other hand, [11] proposed a multi-omics integration strategy using adaptive graph learning and attention mechanism (MOGLAM) that introduced adaptive learning on sample similarity network for each omics and fused the omic-specific representation learning using the multi-omics attention mechanism. Similarly, [6] proposed a multi-omics graph attention network (MOGAT) to improve cancer subtype prediction by integrating eight types of omics data and using a graph attention network (GAT). This method addresses limitations in existing multi-omics integration approaches by leveraging the attention mechanism in GAT to enhance the extraction of significant features from multi-omics data. A patient similarity graph is constructed for each omics data, while patient embedding consists of all eight types of omics expression. The final omics-specific embeddings of the patients were trained using GAT and concatenated to form the final embedding, which was then used for subtype prediction, visualization, and survival analysis.

In addition, some studies incorporate prior biological knowledge from related source domains, such as protein-protein interactions (PPI), as the input graph to further improve the model performance of multi-omics cancer classification. For example, [9] proposed a novel end-to-end deep learning model incorporating prior knowledge and multi-omics data to classify molecular subtypes. Prior knowledge data were used to form a single unified network, and the multi-omics data were concatenated as gene features of the graph, leveraging GCN to learn graph embedding and parallel network as a global feature extractor. In a subsequent study, the authors proposed an enhanced version that integrates multi-omics data in the form of heterogeneous multi-layer graphs, combining both inter-omics and intra-omic connections from prior biological knowledge [8]. In another work, [7] proposed a multiple prior knowledge into graph neural network (MPK-GNN) framework with four main modules. Multi-omics data were concatenated, and sample feature was extracted using a DNN-based sample module, while the GNN-based feature-level module was used to extract features from the prior knowledge graph together with multi-omics data. These studies highlighted the potential of GNN-based frameworks to enhance the analysis and interpretation of multi-omics data by effectively integrating prior biological knowledge. However, gene-based graph models that are based on prior knowledge are challenging as the quality of the extracted features by graph neural network depends on the completeness of the prior knowledge [8, 9].

Besides incorporating prior knowledge, increasing the number of omics data types in integrative modeling is believed to enhance model performance [6]. However, most studies rely on typical early fusion methods, such as concatenation, which fail to account for the varying contributions of each omics data type to the final classification outcome. In contrast, separated feature extractors are utilized for late fusion methods, mainly based on deep neural networks. Nonetheless, this approach increases computational demands as the total input features grow parallel with the number of omics data types. This is evident in the MOGAT model [6], where the experiments were conducted with expensive eight NVIDIA A100 GPUs with large 40GB of GPU memory to handle the integration of eight omics types to the model. In terms of biomarker discovery, some existing studies [5, 10] use feature ablation studies and select potential biomarkers based on feature importance score. The feature importance score is calculated by evaluating the accuracy degradation when the feature is eliminated from the proposed model. Similarly, the discovery becomes challenging when the number of features, such as genes, increases due to more omics data types. Therefore, there are studies [11, 20] that use attention mechanisms or integrated gradients to select the biomarkers as it can avoid gene explosion when the number of genes increases. However, these methods do not consider the gene-gene interactions when ranking the genes. Due to complex human biological gene interaction, some genes might not appear important individually but could play a crucial role when interacting with other genes. This limitation poses a significant challenge when using methods that rank genes solely based on their individual importance scores.

To address the aforementioned limitations stated above, this study proposes a new method called **A**ssociative **M**ulti-**O**mics **G**raph **E**mbedding **L**earning (**AMOGEL**). The main contributions of AMOGEL are summarized as follows:

- Adopting the association rule mining (ARM) model as early fusion multi-omics integrative analysis to mine relationship between the inter-omics datasets, forming information-based multi-omics graph before model training, since early fusion with inter-omics analysis can remove noise (irrelevant genes) before model training.
- Proposing multi-dimensional edges graph with ARM information-based content graph as the main contributor, while the prior knowledge graph act as auxiliary contributor.
- Introducing the gene ranking with consideration of gene-gene interaction contribution by using gene-gene edges attention score.

## Methods

**Fig. 1:**
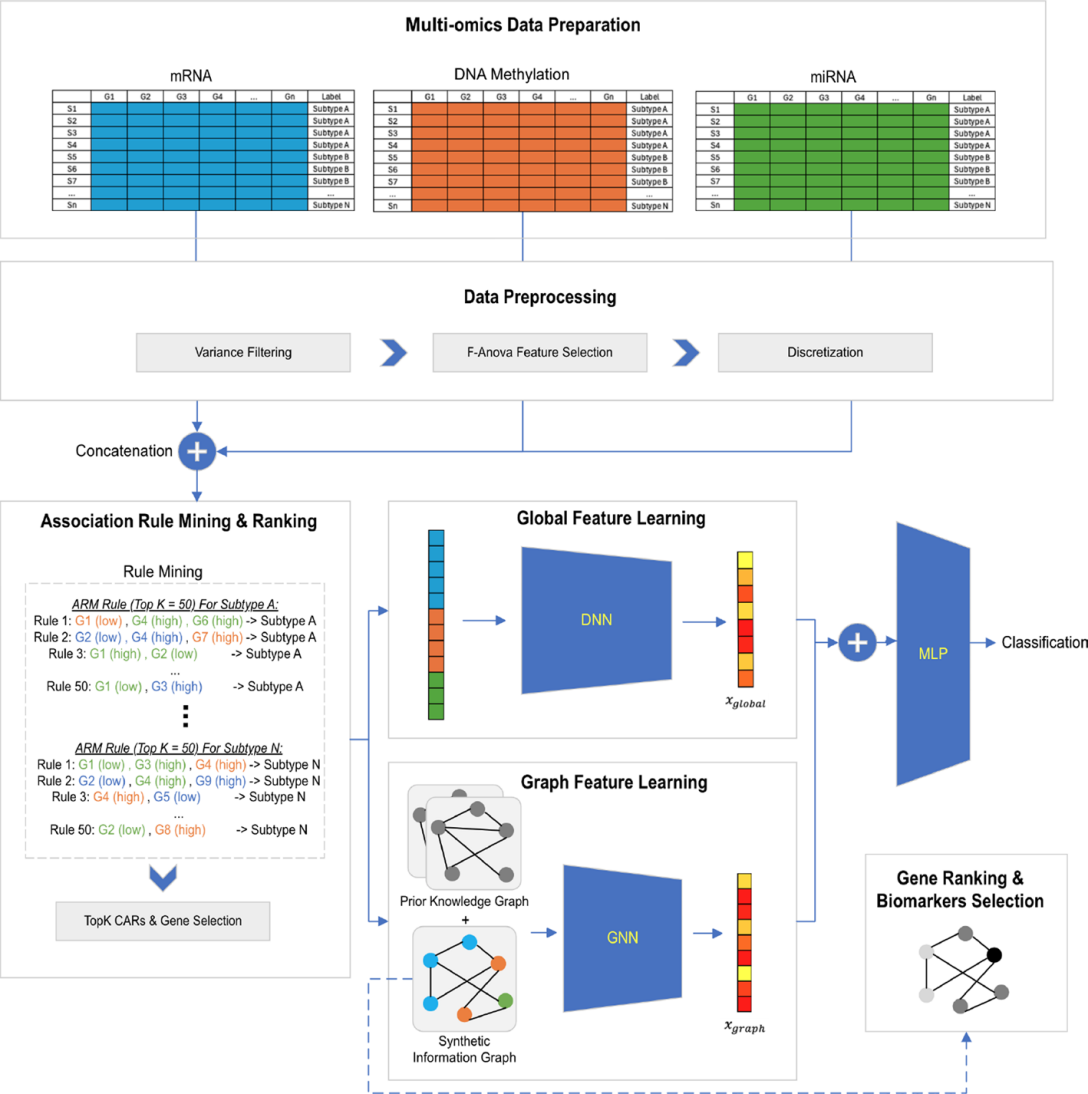
The overall framework of the proposed associative multi-omics graph embedding learning (**AMOGEL**)

The proposed AMOGEL method can be divided into seven parts: (i) data preparation, (ii) data preprocessing, (iii) classification association rule mining and ranking, (iv) graph feature learning, (v) global feature learning, (vi) cancer subtype classification and (vii) gene ranking and biomarkers selection. Fig.  1 shows the overview architecture of our proposed method - AMOGEL. The details of each process will be discussed in the subsequent sections.

### Data preparation

The same datasets from  [11] were used to evaluate the performance of our proposed method - AMOGEL. Cancer subtypes in the breast invasive carcinoma (BRCA) and the pan-kidney (KIPAN) datasets were downloaded from the TCGAbiolink (*https://bioconductor.org/packages/release/bioc/html/TCGAbiolinks.html*) and Broad GDAC Firehouse (*https://gdac.broadinstitute.org/*), respectively. Moreover, three omics data types for each dataset, namely miRNA expression, mRNA expression and DNA Methylation, were chosen due to their highly correlated samples among multiple omics data types. Apart from these multi-omics data, prior biological knowledge, including the KEGG pathway, Gene Ontology (GO) and protein-protein interaction (PPI) networks, are integrated into our study to further improve the model. PPI networks provide the interaction between protein genes with interaction confidence scores based on evidence. Meanwhile, the KEGG Pathway is a collection of biological pathways representing molecular interactions within cells, while the Gene Ontology is the vast knowledge database that unifies the representation of gene and gene product attributes across multiple species, including humans. The PPI dataset was downloaded from the STRING database [21], while the KEGG pathway and GO were retrieved from Database for Annotation, Visualization and Integrated Discovery (DAVID) [22, 23].

### Data preprocessing

Retrieved multi-omics datasets are highly noisy and consist of redundant genes. For BRCA, the mRNA dataset consists of 1212 samples with 20531 genes, the DNA methylation dataset has 885 patient samples with 20106 genes, and the miRNA consists of 1189 patient samples with 503 features. While for KIPAN, the mRNA dataset has 1020 samples with 20531 genes, the DNA methylation dataset consists of 867 patient samples with 20116 genes, and the miRNA is made up of 1005 patient samples with 472 features. Considering the large number of features or genes in the datasets, removing irrelevant and redundant ones is essential to ensure better model interpretation and performance. First, genes with missing values were filtered out and duplicated genes and samples were aggregated before the final mean values were calculated. To correlate each sample across different omics datatypes, the datasets were cross-checked to filter out those that do not exist in these three types of omics datasets. Besides, low-variance genes whose gene expressions do not reflect any changes to different classes were removed from the datasets. A variance threshold of 0.001 was used to filter low-variance genes for mRNA, miRNA and DNA Methylation.

In addition to that, the omics data may still have non-significant features that might result in poor classification performance. Thus, the ANOVA F-value statistical test was used to select only statistically significant features for each omic. Based on the works in [5, 11], to avoid selecting only highly correlated features and ignoring complementary information from less relevant features, the first principle component of the data after feature selection should explain $<50\%$ of the variance. Additionally, the best-performing accuracy among different numbers of input features per omics type is evaluated and selected by using the proposed method. Table 1 summarizes the KIPAN and BRCA datasets, the number of samples for each cancer subtype, and the number of genes before and after the data preprocessing.

**Table 1:** Summary of the multi-omics datasets

Dataset	Subtypes and number of samples after data preprocessing	Number of features in mRNA, DNA Methylation and miRNA	Number of features after data preprocessing^a^
BRCA	Normal-like: 115 Basal-like: 131 HER2-enriched: 46 Luminal A: 546 Luminal B: 147	20531, 20106, 503	1000, 1000, 502
KIPAN	KICH: 66 KIRC: 318 KIRP: 273	20531, 20111, 472	2000, 2000, 471

^a^To ensure the fair comparison, ANOVA-F filtering threshold for BRCA is 1000 and KIPAN is 2000, same threshold reported in MOGONET [5] and MOGLAM [11]

### Classification association rule mining and ranking

Due to the complex biological systems, a gene network generated using the prior knowledge (PPI/KEGG pathway/GO) is inadequate to represent the complex human biology system [8, 9]. Therefore, in this research, we proposed to use the association rule mining technique to mine intra-omics and inter-omics gene association interaction as an early fusion technique. The generated class association rules (CARs), which consist of genes and the cancer subtypes, were subsequently used to form the gene network as the main contributor in our proposed classification model. These generated rules are easily interpretable, making this method widely preferred across various fields, such as market basket analysis [24], to identify associations among items that are frequently purchased together.

Association rule mining (ARM) is a technique that is used to discover interesting patterns, relationships, and associations among a set of variables in large datasets. The goal of ARM is to identify rules that describe the co-occurrence of items in the transaction database. The generated rules can be expressed in the form of “if A, then B” where A is called antecedent, and B is called consequent. To discover association rules, ARM finds frequent item sets from large transaction databases by counting the frequency of occurrence of a particular item set. Those items that are more than *minimum support* are defined as frequent itemsets. *Minimum confidence* user-defined threshold was also used to measure the strength of an association rule. The support and confidence of the rules can be calculated using the following conditional probability expressed as Eq. 1 and Eq. 2 below:

1 $$\begin{aligned} & support(A \Rightarrow B) = P (A \cup B); \end{aligned}$$

2 $$\begin{aligned} & \quad confidence(A \Rightarrow B) = P(B|A). \end{aligned}$$

Association classification (AC), on the other hand, combines elements of both classification and association rule mining. In AC, the antecedent of the rules will be the itemsets from the transaction database, and the consequent is the class label.

Instead of the late fusion method, mRNA, DNA Methylation and miRNA features were concatenated into a single view before model learning, as compared to other related work [6, 10, 11], which integrated multi-omics by combining the results generated from different omics-specific models. First, the feature columns of multi-omics datasets, mRNA, DNA methylation and miRNA, were concatenated across the row with the same patient index. The combined feature columns were mapped with unique identifiers to uniquely identify the overlapping gene names that exist in mRNA and DNA methylation. As a result, the BRCA dataset has a total of 985 samples and 2502 multi-omics features, while the KIPAN dataset has a total of 657 samples and 4471 multi-omics features, as shown in Table 1. The datasets were then split into 70% training samples, and 30% testing samples, and the expression value of each feature was discretized into either a low, medium or high value based on the distribution value of each feature from the training dataset. The discretization process is also applied to the test dataset based on the feature distribution from the training dataset.

From existing studies [17, 25, 25–29], ARM typically generates large frequent itemsets, especially in high-dimensional gene expression data. Thus, this study proposed an iterative minimum support search algorithm (see Algorithm 1) to generate a minimum set of closed frequent item sets [30] for each class subset. Transaction databases were generated based on the class output of each sample and cancer subtype, and the initial minimum support value was calculated for each transaction database based on the total number of transactions. By using the initial minimum support value, a list of frequent itemsets was generated using the ARM technique. This process was repeated by decreasing the minimum support value until the generated list of frequent itemsets passed the maximum rules count. By doing so, it helps to prevent class rules imbalance due to imbalance class from the original datasets. The generated frequent itemsets represent the antecedents, while their respective class labels represent the consequences of the generated CARs. Next, weak CARs were filtered out using the $minimum\;confident\;threshold$.

**Algorithm 1:**
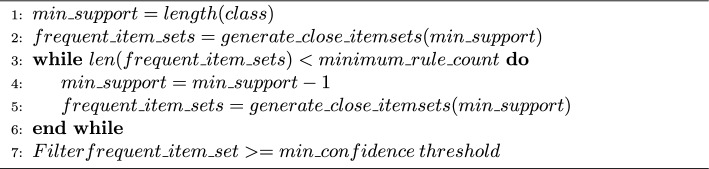
Iterative minimum support search

The generated CARs that met the user-defined threshold *min_support* and *min_confidence* are considered strong rules. However, strong rules are not necessarily interesting. In order to rank the rules based on their interestingness, modified information-content measurement [14, 31] was used, which is expressed in Eq. 3. In the equation, confidence was added to the interestingness ranking to further enhance the quality of the ranked rules. Top generated CARs (top-*k*) from each class subtype were filtered, and the *k* threshold in this study was set to 1000. The Top-*k* CARs were then used for the graph feature learning and global feature learning, which are detailed in the subsequent subsections.

3 $$\begin{aligned} \operatorname {information-content} = log_2(\operatorname {information\,gain}) + log_2(\operatorname {correlation}) + log_2(\operatorname {confidence}). \end{aligned}$$

### Graph feature learning

Generally, a network graph can be represented using the notation $G=\{V,E\}$, where *V* is the set of genes, and *E* is the edge between genes. For the proposed AMOGEL, a synthetic information graph was generated from the information-content-based top-*k* CARs, and prior knowledge graphs were generated from prior biological knowledge gathered from the PPI, KEGG pathway and GO databases. Both types of graphs were unified to form the final static graph.

#### Synthetic information graph

**Fig. 2:**
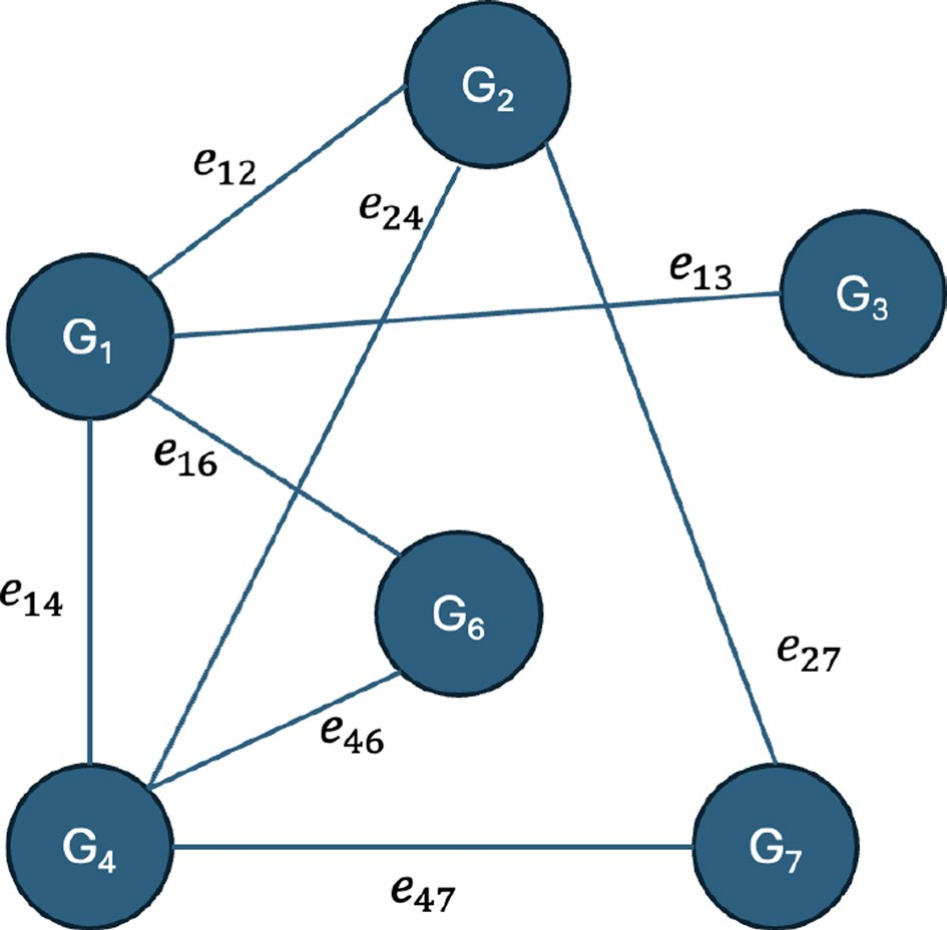
Information graph. The edge between the node $G_i$ and node $G_j$ is connected if the information-content of both $G_i$ and $G_j$ is more than a certain threshold

Distinct genes that exist from the selected top-*k* CARs were represented as nodes of the graph. The information-content between genes was represented by an edge between nodes. Given gene *i* and gene *j*, the edge between genes can be defined using Eq. 4:

4 $$\begin{aligned} e^{information}_{ij} = \frac{infogain_{i}+infogain_{j}+correlation_{i}+correlation_{j}}{4}, \end{aligned}$$

where the $infogain_i$ is defined as mutual information between gene *i* and the cancer subtypes, while the $correlation_i$ is the correlation between gene *i* and the class. The gene *i* and gene *j* belong to any specific rule R within the set of CARs, $\{i,j\} \subseteq R, \quad R \in \textit{CARs}$. The adjacency matrix for the synthetic information graph $A^{information}_{ij} \in \mathbb {R}^{m \times m}$ can be constructed using $A_{ij} = e_{ij}$, where *m* is the number of selected genes from the top-*k* CARs, as shown in Fig. 2. Less informative edges between nodes were filtered out with a threshold set to 0.3. This helps to reduce the noise contributed to model training and to improve model training time due to a decrease in graphs’ sparsity but without compromising the model accuracy as denoted in Eq. 5:

5 $$\begin{aligned} A^{information}_{ij}={\left\{ \begin{array}{ll} e_{ij}, & \text {if} \,\, e_{ij}> \,\, \text {threshold} , \{i,j\} \subseteq R, \quad R \in \textit{CARs};\\ 0, & \text {otherwise}. \end{array}\right. } \end{aligned}$$

#### Prior knowledge graph

**Fig. 3:**
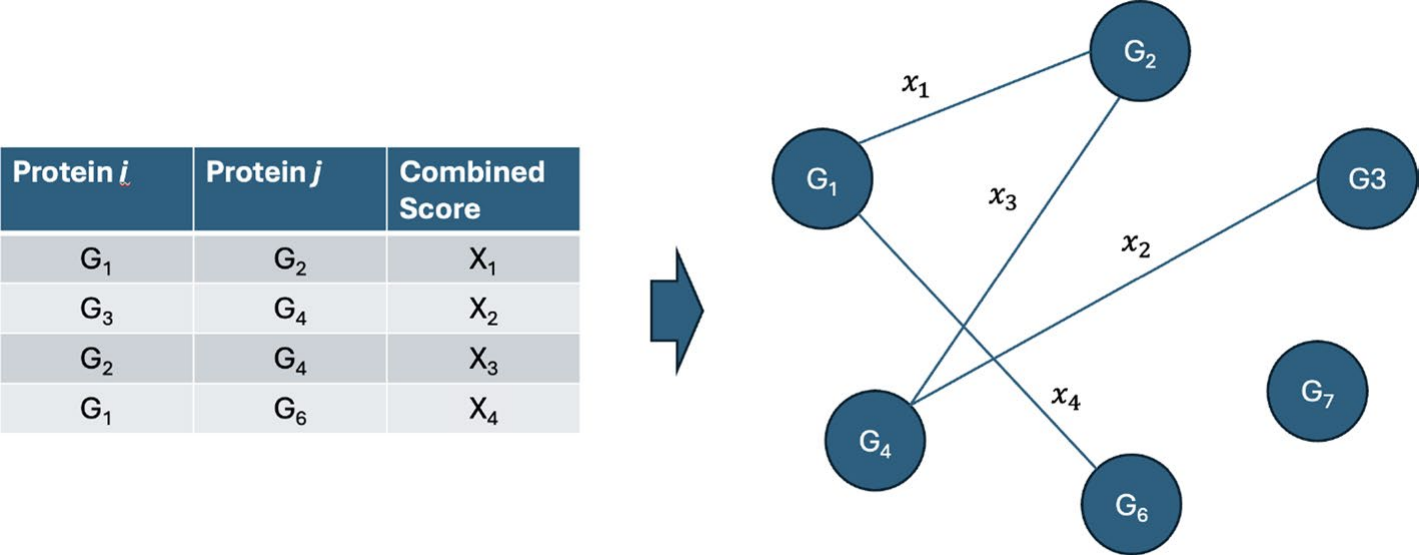
PPI edges construction. The edge between the node $G_i$ and node $G_j$ is connected if there is a PPI pair consisting of both $G_i$ and $G_j$. The attribute used for the edge is the combined score from the PPI database

Apart from the synthetic information graph, prior knowledge information was also injected into the final static graph to further enhance the model performance. One of the benefits of the graph neural network is its ability to learn from non-Euclidean relations, such as prior knowledge graph information. The PPI table obtained from the STRING database consists of two gene columns, each representing a gene involved in the interaction, along with a corresponding confidence score indicating the reliability or strength of the interaction between genes. For this study, protein-protein interaction with a confidence score equal to or more than 500 was selected to prevent weak protein gene interaction from being added to the final graph. Each set of genes was used to construct the gene network graph as shown in Fig.  3. By using the same gene set extracted from the synthetic information graph, an edge between gene *i* and gene *j* is connected if the genes exist in PPI prior knowledge and the confidence score is used as the attribute value for the edge, which can be denoted as $e^{ppi}_{ij}$, as shown in Eq. 6. Due to the PPI database being limited to the protein-gene network, there will be no edges between the selected miRNA nodes and miRNA-mRNA nodes from the top-*k* CARs. In other words, identical genes across inter-omics datasets will have an edge connecting each other if the source and target genes exist in the PPI network. The final normalized adjacency matrix for the PPI graph, $A^{ppi}_{ij} \in \mathbb {R}^{m \times m}$, can be constructed as $A^{ppi}_{ij} = normalize(e^{ppi}_{ij})$, value range from 0 to 1, *m* is the number of selected genes from the top-*k* CARs.

6 $$ e_{{ij}}^{{ppi}}  = \left\{ {\begin{array}{*{20}l}    {confidence\_score_{{ij}} ,} \hfill & {{\text{if}}\,\,{\text{confidence\_score}}\,{\text{ > }}\,{\text{500}}} \hfill  \\    {0,} \hfill & {{\text{otherwise}}} \hfill  \\   \end{array} } \right. $$

**Fig. 4:**
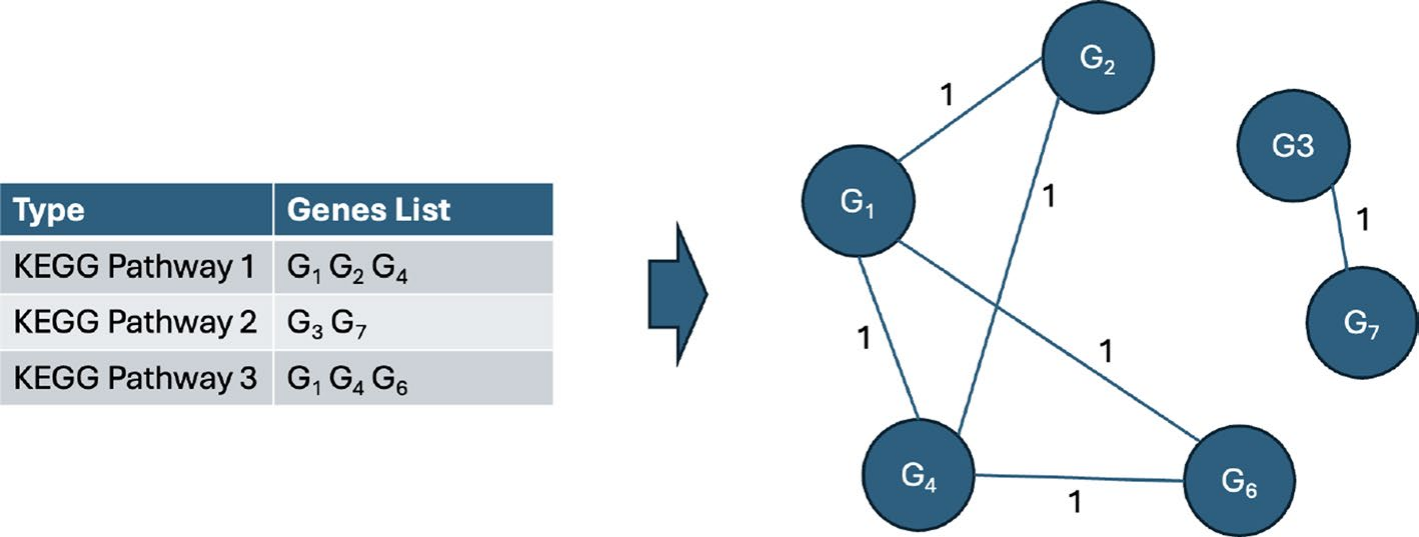
KEGG edges construction. The edge between the node $G_i$ and node $G_j$ is connected with an attribute of 1 if there is a KEGG pathway term consisting both $G_i$ and $G_j$

**Fig. 5:**
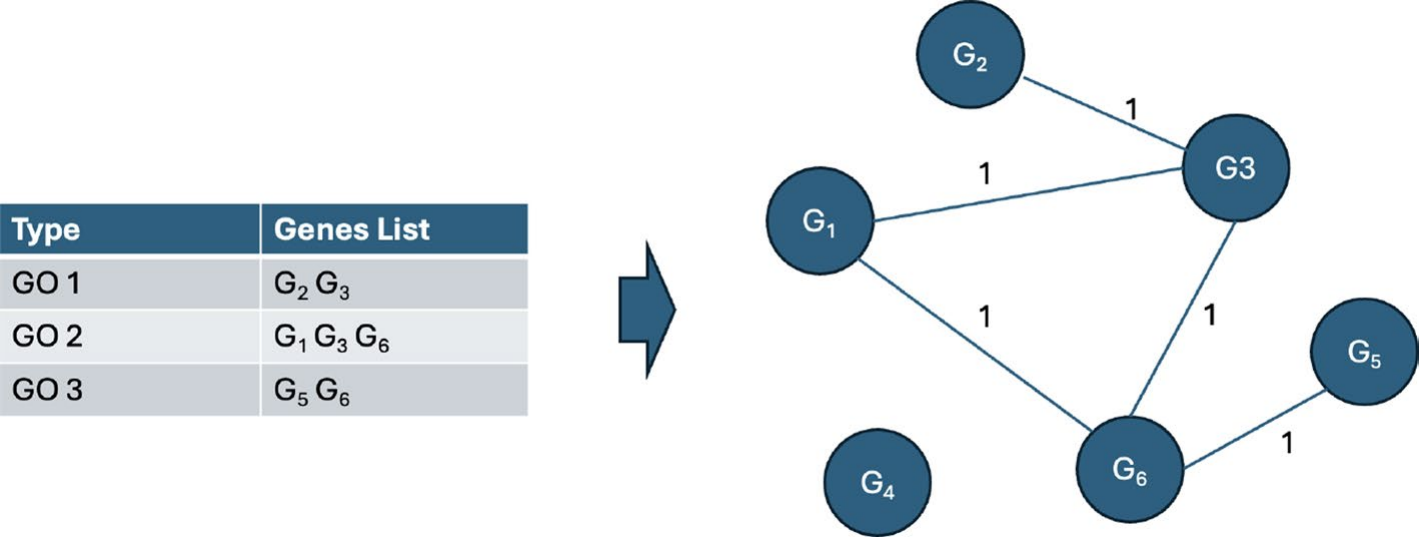
GO edges construction. The edge between the node $G_i$ and node $G_j$ is connected with an attribute of 1 if there is a GO term consisting both $G_i$ and $G_j$

Apart from that, two functional enriched graphs were constructed using the KEGG pathway and GO obtained from the DAVID database. It provides valuable information on the involvement of genes in specific biological pathways and their functional roles according to Gene Ontology terms. The functional annotation chart obtained from DAVID provides a comprehensive overview of the association between pathways or Gene Ontology terms and their related genes. In the KEGG pathway annotation chart, each entry includes a specific pathway term along with a list of genes associated with that pathway. For the GO annotation chart, each entry encompasses a Genome Ontology term, and the corresponding genes linked to that specific biological process, cellular component, or molecular function are detailed. Related genes for each KEGG pathway and GO term were used to construct the KEGG pathways graph and GO genes graph, as shown in Figs. 4 and 5. The edge between gene *i* and gene *j*, $e^{kegg}_{ij}$ and $e^{go}_{ij}$ defined in Eq. 7, is constructed with an attribute value of 1 if there is a KEGG pathway or GO term consist of both gene *i* and gene *j*. Similar to PPI, the KEGG pathway and GO terms with a significant value of less than 0.05 were filtered out. Adjacency matrix for KEGG pathway graph, $A^{kegg}_{ij} \in \mathbb {R}^{m \times m}$, and GO graph, $A^{go}_{ij} \in \mathbb {R}^{m \times m}$, can be constructed using $A^{kegg}_{ij} = e^{kegg}_{ij}$ and $A^{go}_{ij} = e^{go}_{ij}$.

7 $$\begin{aligned} e_{{ij}}^{{kegg}} & = \left\{ {\begin{array}{*{20}l} {1,} \hfill & {{\text{if }}\,{\text{there}}\,{\text{ is }}\,{\text{KEGG}}\,{\text{ pathway}}\,{\text{ term}}\,{\text{ consist}}\,{\text{ of}}\,{\text{ both }}\,{\text{gene}}\,i\,{\text{and}}\,j.} \hfill \\ {0,} \hfill & {{\text{otherwise}}.} \hfill \\ \end{array} } \right. \\ e_{{ij}}^{{go}} & = \left\{ {\begin{array}{*{20}l} {1,} \hfill & {{\text{if }}\,\,{\text{there}}\,{\text{ is}}\,{\text{ GO}}\,{\text{ term}}\,{\text{ consist}}\,{\text{ of}}\,{\text{ both }}\,{\text{gene}}\,i\,{\text{ and}}\,j.} \hfill \\ {0,} \hfill & {{\text{otherwise}}.} \hfill \\ \end{array} } \right. \\ \end{aligned}$$

#### Final static graph

With the synthetic information graph and prior knowledge graphs, a final static graph $G^{final}$ can be constructed. Compared to other existing works that solely used prior knowledge to generate the gene networks, we proposed an information-based graph as the main contributor, while prior knowledge was used as the auxiliary contributor. For the final edge construction for $G^{final}$, we proposed the use of multi-dimensional edges to retain as much information from both the information graph and prior knowledge graphs. To our knowledge, this is the first time that the gene graph has been constructed using multi-dimensional edges for cancer subtype classification. The final edges adjacency matrix $A^{final}$ can be represented as in Eq. 8:

8 $$\begin{aligned} {\begin{matrix} A^{final}&= stack[ A^{information} , \lambda A^{ppi} , \lambda A^{kegg} , \lambda A^{go} ], \end{matrix}} \end{aligned}$$

where the $\lambda$ is the mean value from $A^{information}$, $A^{final} \in \mathbb {R}^{m \times m \times 4}$. Since deep learning models tend to be influenced by higher values and learn from large weighted edges, the edge attribute of PPI, KEGG pathway and GO were scaled by $\lambda$ to avoid large contributing factors from prior knowledge in the model training.

#### Graph neural network

Once the unified gene graphs were constructed, the graphs were parsed into the graph neural network model. Given our final input graph $G^{final}$, along with a set of node features $X\in \mathbb {R}^{m \times 3}$ and edge information in adjacency matrix $A^{final} \in \mathbb {R}^{m \times m \times 4}$, *m* is the number of selected genes, the initial node embedding for the input graph is one-hot encoded (low, medium, and high). Information is aggregated from $\mu$’s node graph neighbourhood $N(\mu )$ for each node $\mu \in V$ and hidden embedding $h_{\mu }^{(k+1)}$ of the $\mu$ is updated together with the current state of $\mu$’s hidden embedding $h_{\mu }^{(k)}$, which can be expressed using the Eq. 9:

9 $$\begin{aligned} h_{\mu }^{(k+1)} = UPDATE^{(k)} \Big ( h_{\mu }^{(k)} , AGGREGATE^{(k)} ( \{ h_{\nu }^{(k)} , \forall \nu \in N(\mu ) \} ) \Big ). \end{aligned}$$

To learn the neighbour embedding, graph attention convolution (GATConv) [32] was used as the technique for neural message passing. This is used to capture the varying importance of the gene’s neighbor and it is also part of the gene ranking method for biomarker selection. The attention coefficients of neighbour genes were learned through the self-attention mechanism, which enables the model to focus on relevant neighbour genes for each node. The neural passing can be represented as the following Eq. 10 and Eq. 11:

10 $$\begin{aligned} h_{\mu }^{(k+1)} = \alpha _{\mu ,\mu }\Theta _{s}h_{\mu }^{(k)} + \sum _{\nu }^{N}\alpha _{\mu ,\nu }\Theta _{t}h_{\nu }^{(k)}, \end{aligned}$$

where the attention coefficients $\alpha _{\mu ,\nu }$ are computed as

11 $$\begin{aligned} \alpha _{\mu ,\nu }=\frac{exp\Big (LeakyReLU\Big (\vec {a}^{T}[W\vec {h}_{\mu } \mathbin \Vert W\vec {h}_{\nu }]\Big )\Big )}{\sum _{k \in N}exp\Big (LeakyReLU\Big (\vec {a}^{T}[W\vec {h}_{\mu } \mathbin \Vert W\vec {h}_{k}]\Big )\Big )},s \end{aligned}$$

where $\cdot ^{T}$ represents transposition and $\mathbin \Vert$ is the concatenation operation.

In AMOGEL, two layers of GATConv message passing are used. For the first layer of GATConv, every node embedding contains information from 1-hop of their neighbourhood. After two layers of GATConv, every node embedding will contain 2-hop of neighbourhood information. Adding more layers of GATConv is not recommended due to the smoothing effect, as the node representations tend to become more similar or “smoothed” across the graph. Apart from that, concatenation and skip connections of each GATConv layer were used to prevent the smoothing effect and improve the model’s ability to capture local and global patterns in the graph, which leads to a deeper neural network. After three layers of GATConv message passing, the gene nodes will be encoded with information of neighbouring features from 2-hops away. A final graph embedding is generated by applying global mean pooling on all the trained node embedding and subsequently is passed into a shallow multi-layers perceptron to learn the final graph embedding representation.

### Global feature learning

As mentioned before, the embedding learned from graph neural networks is highly dependent on the quality of the graph structures. Instead of relying on the graph structure domain, selected *m* gene features from the top-*k* CARs will be used as input to a feedforward deep neural network model. With a deep neural network, global feature representations can be learned independently without considering any gene-gene interactions. To enhance the overall performance of the classification model, the global feature learning module was trained in parallel with the graph feature learning module, as shown in Fig.  1. This approach allows the final classification model to learn both global features and graph features from the selected genes in the top-k CARs.

### Cancer subtype classification

The embedding output from graph feature learning and global feature learning denoted as $\varvec{x}_{graph}$ and ${\varvec{x}_{global}}$ were further concatenated to form lower-dimensional representation and passed through to a multi-layers perceptron to learn the final embedding for classification as shown in Fig.  1.

### Gene ranking and biomarker selection

With the trained model, genes were ranked by averaging attention scores generated using the GATConv message passing for all two layers across all the samples. Based on ranking, higher-ranked genes have higher contributing factors and stronger links to neighbour genes, which contribute more to the final classification. The top 100 ranked genes were further evaluated using the DAVID database to evaluate the relevancy of the selected biomarkers. Given the learned attention coefficients $\alpha _{{\mu },{\nu }}$ for gene $\mu$ and $\nu$ from Eq. 11, gene *i* ranking can be calculated using the following Eq. 12:

12 $$\begin{aligned} Rank(Gene_{i}) = \sum _{j=1}^{m} \left( \frac{\alpha ^{GATConv1}_{ij}+\alpha ^{GATConv2}_{ij}}{2}\right) , \end{aligned}$$

where $\alpha ^{GATConv1}$, $\alpha ^{GATConv2}$ represented attention coefficients from 2 GATConv layers, *m* is the number of selected genes from the top 1000 CARs.

## Results and discussion

### Experiment setup

The KIPAN and BRCA datasets used in this research were split into training and test datasets using a 7:3 ratio. The class distribution for each dataset was equally divided between the training and test datasets. To avoid any bias in data splitting, the experiment was repeated five times with random split sampling. The learning rate was set at 0.00005, and the experiments were run for 500 epochs. The experiments were implemented in Python version 3.9.18, using the PyTorch framework along with the torch-geometric module. The experiments were conducted on a local machine with an Intel Xeon W-2145 processor, 64GB of RAM, and Nvidia Quadro P5000 GPU. For model performance evaluation, the following metrics were used: accuracy, macro F1 score, and macro AUROC. The mean and standard deviation (STD) were recorded for each metric. The accuracy measures the overall correctness of the AMOGEL and be expressed in the following Eq. 13:

13 $$\begin{aligned} Acurracy = \frac{\sum _{i=1}^{C}True\,Positives}{Total\,Samples}. \end{aligned}$$

where *C* is the total number of classes. Apart from accuracy, the macro F1 score was used as a metric to measure the performance of the model, which can be interpreted as following Eq. 14:

14 $$\begin{aligned} {\begin{matrix} Precision_{i} & = \frac{TP_{i}}{TP_{i} + FP_{i} }; \\ Recall_{i} & = \frac{TP_{i}}{TP_{i} + FN_{i}}; \\ F1\,score_{i} & = \frac{2 \times Precision_{i} \times Recall_{i}}{Precision_{i} + Recall_{i}},\\ Macro\;F1\;Score & = \frac{\sum _{i=1}^{C}F1\;score_{i}}{C}; \end{matrix}} \end{aligned}$$

where $TP_{i}$ is true positives for class *i*, $FP_{i}$ is false positives for class *i*, $FN_{i}$ is false negatives for class *i* and $Support_{i}$ is the number of sample for class *i*. The macro F1 score ranges from 0 to 1, and it measures the balance of precision and recall, with 1 as the perfect classification. The area under the receiver operating characteristic (AUROC) curve measures the model’s ability to distinguish the samples’ classes. The higher AUROC suggests a better overall ability of the model to distinguish the class of a sample from other classes.

For multi-class classification, one of the common loss functions, the categorical cross-entropy loss, was computed for the AMOGEL for backward propagation optimization, as shown in Eq. 15:

15 $$\begin{aligned} Categorical\,Cross\,Entryopy\,Loss = -\frac{1}{N}\sum _{i=1}^{N}\sum _{j=1}^{C}y_{ij}log(p_{ij}), \end{aligned}$$

where *C* is the number of classes, $y_{ij}$ is an indicator (0 or 1) of whether sample *i* belongs to class *j*, and $p_{ij}$ is the predicted probability that sample *i* belongs to class *j*. adaptive moment estimation (Adam) optimizer with L2 regularisation of 0.001 was used to update the trainable weight of the model during the backward propagation optimization. This is to prevent any overfitting problem from arising during model training.

### Performance comparison and analysis

The proposed method was compared against a few conventional models, including k-nearest neighbour classifier (KNN), support vector machine (SVM), and naive bayes (NB). Apart from that, we compared the proposed method with a feed-forward deep neural network model (DNN), and the state-of-the-art GNN models (MOGONET [5] & MOGLAM [11]), specifically for cancer subtype classification. For KNN, SVM, NB and DNN models, the preprocessed multi-omics datasets mRNA, miRNA and DNA methylation were concatenated horizontally before the model training and testing. For MOGONET [5] and MOGLAM [11] models, each preprocessed multi-omics dataset was trained with omics-specific classifier, followed by the fusion technique for final classification. The results are shown in Table 2.

**Table 2:** Classification performance on BRCA and KIPAN datasets

Experiment	BRCA			KIPAN		
	Acc (STD)	F1 (STD)	AUROC (STD)	Acc (STD)	F1 (STD)	AUROC (STD)
KNN	72.12(1.6)	45.56(1.1)	79.19(1.5)	93.03(1.6)	92.24(1.5)	96.80(0.9)
SVM	72.47(2.7)	44.39(4.5)	90.56(1.0)	92.89(1.9)	91.86(2.3)	98.45(0.5)
NB	81.39(3.1)	74.87(4.9)	92.50(2.5)	95.86(1.8)	94.58(2.2)	97.21(0.7)
DNN	75.58(2.7)	62.37(5.4)	90.05(1.0)	95.15(1.5)	94.12(2.2)	98.81(0.7)
MOGONET [5]	75.50(2.1)	60.96(5.9)	87.97(2.9)	95.65(1.2)	94.90(1.6)	97.38(1.8)
MOGLAM [11]	76.88(2.3)	64.97(4.1)	92.13(1.0)	94.75(1.5)	93.72(1.4)	98.64(0.6)
AMOGEL	**86.32(1.7)**	**75.67(4.4)**	**94.36(0.6)**	**96.06(1.4)**	**95.08(1.4)**	**99.37(0.4)**

Bold indicates the highest value and the bracket values are the standard deviation

From the result, AMOGEL outperformed other models in terms of accuracy (Acc), F1-score (F1) and AUROC for both BRCA and KIPAN datasets. The BRCA dataset recorded the highest improvement as compared to other existing methods in terms of accuracy and F1 score performance. For kidney cancer subtype classification, the lower improvements were due to the higher interpretability of its omics dataset, where differences among KICH, KIRC and KIRP can be easily differentiated by observation [5]. This also signifies that our proposed method has the ability to outperform other methods and classify much more complex dataset scenarios. Apart from that, we also performed a comparison study with the current state-of-the-art model MPK-GNN [7], integrated with multi-omics data with multiple prior knowledge. For a fair comparison, AMOGEL model performance was evaluated with the BRCA dataset and prior knowledge from the same source as mentioned by MPK-GNN [7] research study. Prior knowledge of gene-gene interaction (GGI), PPI and Co-expression network was used, and the comparison result is shown in Table 3. The result showed that the AMOGEL has better accuracy than MPK-GNN model by 10.4%.

**Table 3:** Classification performance comparison on BRCA dataset with prior knowledge GGI, PPI & Co-expression Network

Model	Accuracy (STD)
MPK-GNN [7]	66.2 (1.0)
AMOGEL	76.6 (2.4)

The accuracy result of MPK-GNN were retrieved from the experiment result with 7:3 data splitting

### Ablation studies

In this study, we conducted ablation studies on the proposed method, which can be separated into three modules, namely: ARM feature selection, graph feature learning, and global feature learning, as shown in Fig.  1. To study the effectiveness of the proposed ARM technique as early fusion, we applied ANOVA-F as a typical feature selection technique to reduce the total number of omics features to be the same as the final total number of omics features using the ARM technique. The total number of omics features after applying the ARM technique is, on average, 450 for the BRCA dataset and 1000 for the KIPAN dataset. For the *FS(Dist1)_DNN* method, each omics data was equally selected using ANOVA-F feature selection, which is 150 mRNA features, 150 DNA Methylation features, and 150 miRNA features for the BRCA dataset, while 333 mRNA features, 333 DNA Methylation features, and 333 miRNA features for the KIPAN dataset. For the *FS(Dist2)_DNN* method, multi-omics data were concatenated, and features were selected using the ANOVA-F feature selection technique. The summary of the total number and distribution of each omics data for the BRCA and KIPAN datasets is shown in Table 4.

For rule pruning in ARM feature selection, we compared our proposed method, fix top-*k* rule pruning based on rule ranking (Top*k*), against the existing ARM rules pruning methods, class-based association (CBA) and dynamic top-*k* rule selection DNN classifier. CBA, which was originally introduced by [33], has been widely used by other researchers due to its effectiveness and efficiency. For DNN, different numbers of top-*k* rules $k={10,20,30,40,50,100,200,300,400,500,1000,1500,2000}$, which were sorted by information-based content ranking, were evaluated using the DNN classifier and the best performing top-k rules DNN classifier was selected. By comparing different classifiers for ARM rule pruning, our proposed method achieved the highest performance compared to the CBA and DNN classifiers, as shown in Table 7. This could be due to the low number of genes selected from the low number of left-over rules after CBA classifier rule pruning. Some of the important genes might be left out of the selected rules. Similar to the CBA classifier, a lower performance from the DNN classifier may be due to the instability of top-*K* selection, which results in varying gene selection in different numbers of trials. Tables  5 and 6 show a summary of the number of selected genes and CARs with different type rule pruning for ARM module for both BRCA and KIPAN datasets.

Apart from that, we conducted an ablation study on the effectiveness of prior knowledge in cancer subtype classification incorporated in graph feature learning module (GNN) and the effectiveness of each integration of DNN module and GNN module in parallel. Table 7 shows the overall ablation study results. Based on the results, the proposed ARM technique, *ARM(Top1000)_DNN*, is proven to be an effective inter-omics fusion strategy by attaining higher accuracy, F1 and AUROC scores for the BRCA and KIPAN datasets when compared to models without ARM, namely *DNN*, *FS(Dist1)_DNN* and *FS(Dist2)_DNN*. By reducing the input features same as *ARM(Top1000)_DNN*, *FS(Dist1)_DNN* and *FS(Dist2)_DNN*, their accuracy and F1 score are still unable to exceed *ARM(Top1000)_DNN* performance. This could be due to mRNA features having more valuable information than DNA methylation and miRNA. The features count, and distribution among omics before input into the DNN model is shown in Table 4, as the ARM feature selection consistently selects more mRNA features for both the BRCA and KIPAN datasets. The result also showed that by using the proposed ARM module as the inter-omics fusion strategy, the method could mine the inter-omics relationship and extract relevant omics features from multi-omics data for subsequent tasks. Besides that, when compared to experiment models with prior knowledge *ARM(Top1000)_GNN(allprior)_DNN* & *ARM(Top1000)_GNN(allprior)* and models without prior knowledge *ARM(Top1000)_GNN(noPrior)_DNN* & *ARM(Top1000)_GNN(noPrior)*, there is an improvement in overall metrics if prior knowledge is integrated in the model for both BRCA and KIPAN datasets. This observation confirms the importance of integrating prior knowledge in the model, as also demonstrated by other studies in the literature [7–9].

**Table 4:** Summary of mRNA, DNA Methylation and miRNA features count and distribution before DNN model

Experiment	BRCA			KIPAN		
	mRNA (%)	DNA (%)	miNRA (%)	mRNA (%)	DNA (%)	miNRA (%)
DNN	1000(40%)	1000(40%)	502(20%)	2000(45%)	2000(45%)	471(10%)
FS(Dist1)_DNN	150(33%)	150(33%)	150(33%)	333(33%)	333(33%)	333(33%)
FS(Dist2)_DNN^a^	230(51%)	217(48%)	3(1%)	435(44%)	554(55%)	11(1%)
ARM(Top1000)_DNN^ab^	188(42%)	157(35%)	98(22%)	589(64%)	304(33%)	28(3%)

^a^ Result was reported from one of the trial of the experiments to compare with other single value reporting experiments. ^b^ It was observed that number of selected mRNA features consistently has higher distribution as compared to DNA Methylation and miRNA

**Table 5:** Number of selected genes and CARs result with different ARM rule pruning for BRCA dataset

ARM rule pruning	Number of selected CARs					Number of selected genes				
	T1	T2	T3	T4	T5	T1	T2	T3	T4	T5
CBA	28	20	18	18	21	56	66	56	45	65
DNN	100	2000	10	1500	100	144	533	73	497	154
Top1000	1000	1000	1000	1000	1000	437	448	402	446	379

5 experiments were carried out with random data splitting, denoted as T1,T2,T3,T4 and T5

**Table 6:** Number of selected genes and CARs result with different ARM rule pruning for KIPAN dataset

ARM rule pruning	Number of selected CARs					Number of selected genes				
	T1	T2	T3	T4	T5	T1	T2	T3	T4	T5
CBA	71	51	56	72	56	582	275	414	310	268
DNN	200	1000	20	200	200	862	1323	407	550	412
Top1000	1000	1000	1000	1000	1000	1031	920	936	815	718

5 experiments were carried out with random data splitting, denoted as T1,T2,T3,T4 and T5

**Table 7:** Ablation study

Experiment	BRCA			KIPAN		
	Acc. (STD)	F1 (STD)	AUROC (STD)	Acc. (STD)	F1 (STD)	AUROC (STD)
DNN^a^	75.58(2.7)	62.37(5.4)	90.05(1.0)	94.15(1.5)	94.12(2.2)	98.81(0.7)
FS(Dist1)_DNN^b^	81.56(3.1)	71.32(3.9)	95.86(1.0)	94.34(1.0)	93.02(1.9)	99.04(0.6)
FS(Dist2)_DNN^c^	77.66(2.1)	66.95(3.3)	90.51(1.5)	94.54(0.7)	93.45(2.9)	98.88(0.5)
ARM(Top1000)_DNN^d^	83.20(2.7)	71.78(4.9)	95.80(0.8)	94.95(2.2)	93.74(2.3)	99.03(0.3)
ARM(Top1000)_GNN(noPrior)^e^	83.03(1.2)	70.29(2.2)	94.26(1.3)	90.91(1.9)	90.92(2.0)	97.17(1.7)
ARM(Top1000)_GNN(allPrior)^f^	83.86(1.6)	74.94(1.3)	94.48(1.3)	95.49(1.4)	93.60(1.9)	97.18(1.3)
ARM(CBA)_GNN(allPrior)_DNN^g^	81.73(3.8)	72.86(9.5)	91.80(3.7)	95.35(0.7)	95.04(1.2)	99.05(0.4)
ARM(DNN)_GNN(allPrior)_DNN^h^	85.37(1.5)	74.39(5.5)	94.55(0.9)	95.35(1.2)	94.21(1.4)	99.14(0.4)
ARM(Top1000)_GNN(noPrior)_DNN^i^	83.29(3.5)	72.10(5.3)	95.46(1.6)	95.45(1.7)	94.32(0.9)	98.94(0.6)
ARM(Top1000)_GNN(allPrior)_DNN^j^	**86.32(1.7)**	**75.67(4.4)**	**95.96(0.6)**	**96.06(1.4)**	**95.08(1.4)**	**99.37(0.4)**

Bold indicates the highest value and the bracket values are the standard deviation

^a^Concatenated multi-omics datasets were passed into the DNN model for final classification

^b^Feature selection using ANOVA-F method for each omics data independently

The final distribution of features between omics is equally distributed. Total concatenated omics features are 450 for the BRCA dataset and 999 for the KIPAN dataset

^c^Multi-omics data were concatenated, and subsequently, the features were reduced and selected using the ANOVA-F method. The final number of selected features is 450 for the BRCA dataset and 1000 for the KIPAN dataset

^d^Multi-omics features were selected and concatenated using the ARM technique with Top-1000 ranked CARs. Selected features were passed into the DNN model for final classification

^e^Multi-omics features were selected and concatenated from Top-1000 ranked CARs, generated using ARM technique. The synthetic information graph was constructed for final classification

^f^Multi-omics features were selected and concatenated from Top-1000 ranked CARs, generated using ARM technique. Final static graphs were constructed from the information-based graph and prior knowledge graphs for graph feature learning. Graph feature learning and global feature learning were utilized for the final classification

^g^Multi-omics features were selected and concatenated from pruned CARs by the CBA method. Final static graphs were constructed from the information-based graph and prior knowledge graphs for graph feature learning. Graph feature learning and global feature learning were utilized for the final classification

^h^Multi-omics features were selected and concatenated from the best Top-K CARs based on DNN classifier performance. Final static graphs were constructed from the information-based graph and prior knowledge graph for graph feature learning. Graph feature learning and global feature learning were utilized for final classification

^i^Single dimensional edge graph for AMOGEL model, without prior knowledge information

^j^The proposed AMOGEL method

### Biomarker discovery and interpretation

From the experiment, attention scores on edges were learned by 2 layers of GAT convolution and the best-performing model from five trials was selected and used to generate the candidate gene biomarkers based on the ranking on Eq. 12. Table 8 show the Top 10 ranking result.

**Table 8:** Top 10 ranked genes for BRCA and KIPAN datasets with omics type

Ranking	BRCA		KIPAN	
	Feature	Omics Type	Feature	Omics Type
1	miR-934	miRNA	miR-126	miRNA
2	*RAET1L*	mRNA	*TSPAN5*	mRNA
3	*FOXC1*	mRNA	*VEGFA*	mRNA
4	*ESR1*	mRNA	miR-122	miRNA
5	*MIR563*	DNA Meth.	*UBE2N*	DNA Meth.
6	*FOXA1*	mRNA	*MTUS1*	mRNA
7	*C6orf97*	mRNA	*PLVAP*	mRNA
8	*AGR3*	mRNA	*PRDM16*	mRNA
9	*GATA3*	mRNA	*SLC22A23*	mRNA
10	*TBC1D9*	mRNA	*SNORB30*	DNA Meth.

From literature search, gene *FOXC1* is highly linked to Basal-like subtype and often shows overexpression in Basal-like cancers [34]. It is a transcription factor that regulates the expression of genes involved in cell growth, differentiation and survival. genes *ESR1* and *GATA3* work together in breast cancer, particularly in Luminal subtypes, to regulate gene expression in response to estrogen and drive the growth of estrogen receptor-positive tumors [35, 36]. *FOXA1* gene also facilitates the binding of *ESR1* to its target genes [37], and *GATA3* works with *ERS1* to regulate the transcription of genes that drive the growth and survival of Luminal breast cancer cells. Although gene *C6orf97* is not well characterized, it is located near the *ESR1* gene, and it may be involved in breast cancer susceptibility [38]. *ARG3* gene are often associated with estrogen receptor-positive breast cancer [39] and miR-934 promotes breast cancer metastasis by regulation of *PTEN* and epithelial-mesenchymal transition [40]. *TBC1D9* is also an important modulator of tumorigenesis in breast cancer. [41].

For the KIPAN dataset, miR-126 is known to be downregulated in kidney cancer, including KIRC [42]. It is involved in tumour progression and metastasis while *VEGFA* gene is frequently overexpressed in KIRC and is a target for anti-angiogenic therapies in kidney cancer [43]. miR-122 is also often downregulated in kidney cancer and influences tumour progression by regulating metabolic pathways [44]. Genes *MTUS1* [45], *PLVAP* [46], *PRDM16* [47] and *SLC22A23* [48] are also often dysregulated in kidney cancer, contributing to various aspects of tumour development and progression based on the existing studies. From the biomarkers list, *RAET1L* and MIR563 have limited information in publication that relates to breast cancer, but these two genes are highly ranked (second and fifth), and they may be worth studying to establish a clear connection to breast cancer, similar to how genes *TSPAN5*, *UBE2N* and *SNORD30* for KIPAN datasets are related to pan-cancer disease. The summary of the literature search for the top 10 selected biomarkers was summarized in Table 9.

**Table 9:** Literature search summary for top 10 selected biomarkers

Cancer type	Biomarker	Omics type	Summary
BRCA	mir934 [40]	miRNA	Promotes breast cancer metastasis by regulating PTEN and epithelial-mesenchymal transition.
BRCA	FOXC1 [34]	mRNA	Highly linked to Basal-like subtype, often overexpressed in Basal-like cancers. Regulates genes involved in cell growth, differentiation, and survival.
BRCA	ESR1 [35, 36] GATA3 [35, 36]	mRNA	Work together in Luminal subtypes to regulate gene expression in response to estrogen and drive the growth of estrogen receptor-positive tumors.
BRCA	FOXA1 [37]	mRNA	Facilitates ESR1 binding to its target genes, aiding transcriptional regulation in Luminal breast cancer cells.
BRCA	C6orf97 [38]	mRNA	Not well-characterized; located near ESR1 and may influence breast cancer susceptibility.
BRCA	AGR3 [39]	mRNA	Associated with estrogen receptor-positive breast cancer.
BRCA	TBC1D9 [41]	mRNA	An important modulator of tumorigenesis in breast cancer.
BRCA	RAET1L	mRNA	Limited publication information; highly ranked biomarkers worth investigating for potential breast cancer connections.
BRCA	MIR563	DNA Meth.	Limited publication information; highly ranked biomarkers worth investigating for potential breast cancer connections.
KIPAN	mir126 [42]	miRNA	Downregulated in kidney cancer, including KIRC. Involved in tumor progression and metastasis
KIPAN	VEGFA [43]	mRNA	Frequently overexpressed in KIRC, targeted by anti-angiogenic therapies.
KIPAN	mir122 [44]	miRNA	Often downregulated in kidney cancer, influences tumor progression by regulating metabolic pathways.
KIPAN	MTUS1 [45] PLVAP [46] PRDM16 [47] SLC22A23 [48]	mRNA	Dysregulated in kidney cancer, contributes to tumor development and progression.
KIPAN	TSPAN5	mRNA	Limited publication information; highly ranked biomarkers worth investigating for potential breast cancer connections.
KIPAN	SNORB30 UBE2N	DNA Meth.	Limited publication information; highly ranked biomarkers worth investigating for potential breast cancer connections.

Ranked genes for each dataset were compared with the genetic association database (GAD) disease from the DAVID database [23] as shown in Table 10.

**Table 10:** Biomarkers list associated with breast cancer & kidney cancer from DAVID GAD disease database

Dataset	Term	Genes list	*P* Value	Fold enrich.	FDR
BRCA	Breast Cancer	*BCL2, BLM, BRCA1, POLQ,* ***GATA3***, *RAD51, RAD54B, ZWINT, CHST3, CENPF,* ***ESR1***, *EXO1,* ***FOXA1***, *mir125b2*	1.9e–5	4.0	1.3e–2
BRCA	Breast Cancer	*BCL2, BLM, BRCA1, BUB1,* ***GATA3***, *NDC80, RAD51, TPX2, ASPM, CLSPN,* ***ESR1***, *MCM6, PLK4*	2.9e–4	3.3	5.0e–2
KIPAN	Renal	*CD93, HNF1B, MYCT1, NEK11, FLT1, KDR, LNX1, MGP, NPY, NOTCH4, PTGER3, PRKAG2,* ***VEGFA***	1.9e–2	2.0	6.5e–2

From the associated biomarkers list, the top 10 ranked biomarkers were highlighted in bold. The top 200 ranked genes were compared with the associated disease (breast cancer and kidney cancer) from the DAVID database, and the related biomarkers were listed with fold enrichment and false discovery rate (FDR). Fold enrichment is a measure of how frequently a particular event occurs in a test set compared to a control set, while FDR represents the expected portion of false positives among the declared significant results. Fold enrichment of 4.0 with FDR 1.3% indicates a strong enrichment (four times more frequent in the test set) and a relatively low FDR, suggesting that only 1.3% of the significant findings were expected to be false positive. This also means that this discovered biomarkers list is strong and reliable, indicating a meaningful and trustworthy association. For the second list of detected genes for the BRCA dataset, although it is slightly less than the first biomarkers list, both discovered biomarkers lists were considered significant and reliable, which also demonstrates that the AMOGEL can rank and select the genes accordingly.

To further assess the reliability of identified biomarkers, we conducted external validation using independent datasets obtained from NCBI GEO [49]. The statistical analysis, performed using ANOVA-F tests, confirmed the significance of the top 10 selected biomarkers in distinguishing between subtypes. Table 11 summarize the datasets used, the omic types, subtype sample distributions, and the tested biomarkers.

**Table 11:** Summary of external datasets used for biomarker validation across omics types and BRCA and KIPAN cancer subtypes

Dataset	Omic type	Sample count per subtype	Tested biomarkers	*p* value
GSE1992	mRNA	LumA(34), LumB(19), HER(12), Basal(14), Normal(5)	RAET1L	3.9045e-02
GSE19783	miRNA	LumA(41), LumB(12), HER (17), Basal (15), Normal(10)	miR-934	3.1204e-05
GSE70567	DNA Methy.	LumA(41), LumB (30), HER (32), Basal(44), Normal (11)	MIR563	2.9372e-05
GSE20685	mRNA	LumA(71), LumB(52), HER(51), Basal(31), Normal(39)	GATA3	6.2580e-60
			FOXC1	8.6821e-54
			TBC1D9	1.3633e-45
			ESR1	1.0325e-03
			C6orf97	5.2774e-49
			FOXA1	6.3436e-74
			AGR3	2.9687e-59
GSE15641	mRNA	KIRP(11), KIRC(32), KICH(6)	TSPAN5	2.0067e-08
			MTUS1	1.7907e-05
			PLVAP	2.7228e-06
			VEGFA	5.5400e-07
			PRDM16	7.9022e-03
GSE48008	miRNA	KIRP(4), KIRC(5), KICH(27)	miR-122	9.8094e-01
			miR-126	3.9823e-01

**Fig. 6:**
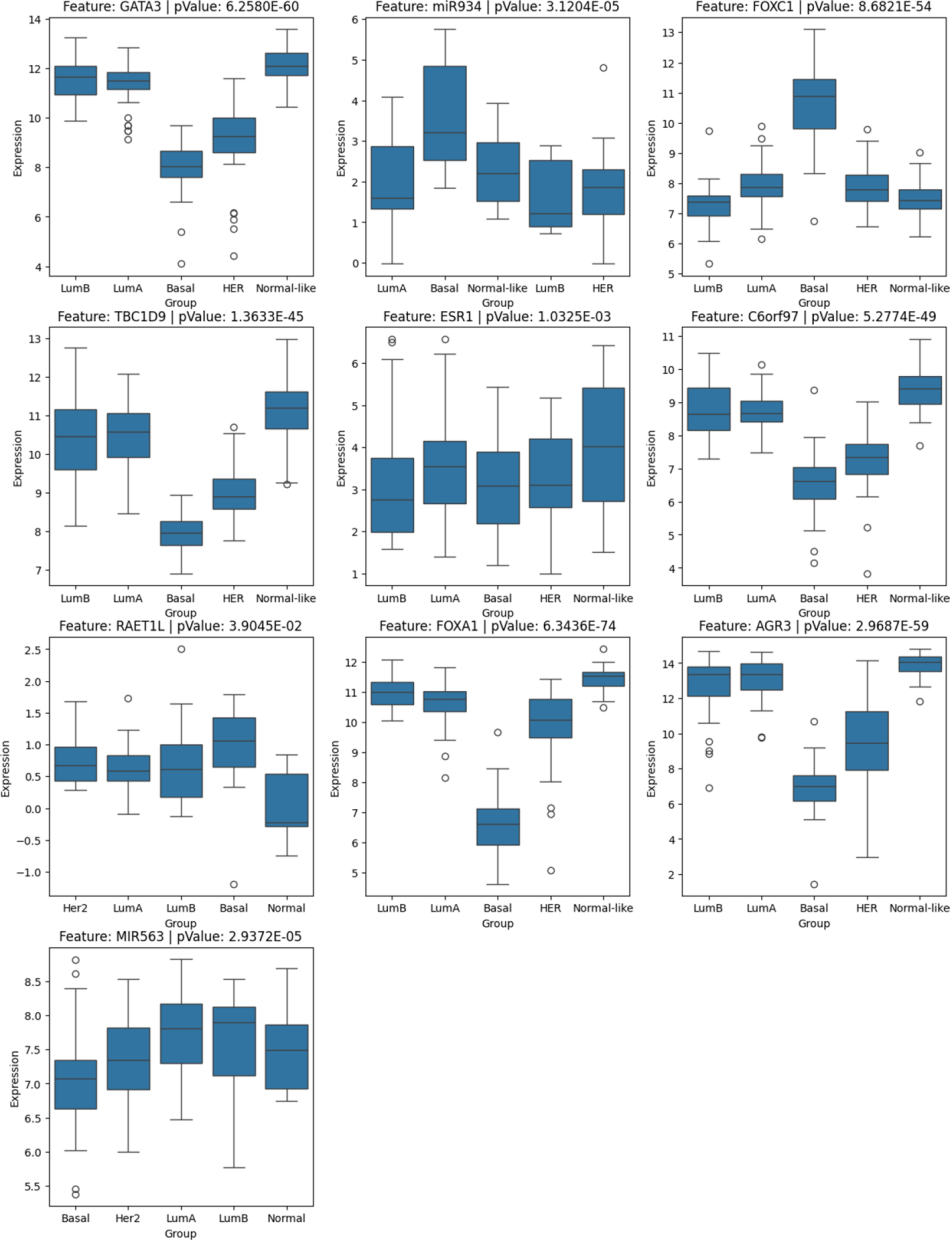
Boxplots showing the expression levels of selected biomarkers across breast cancer subtypes, with corresponding *p*-values from ANOVA-F tests

**Fig. 7:**
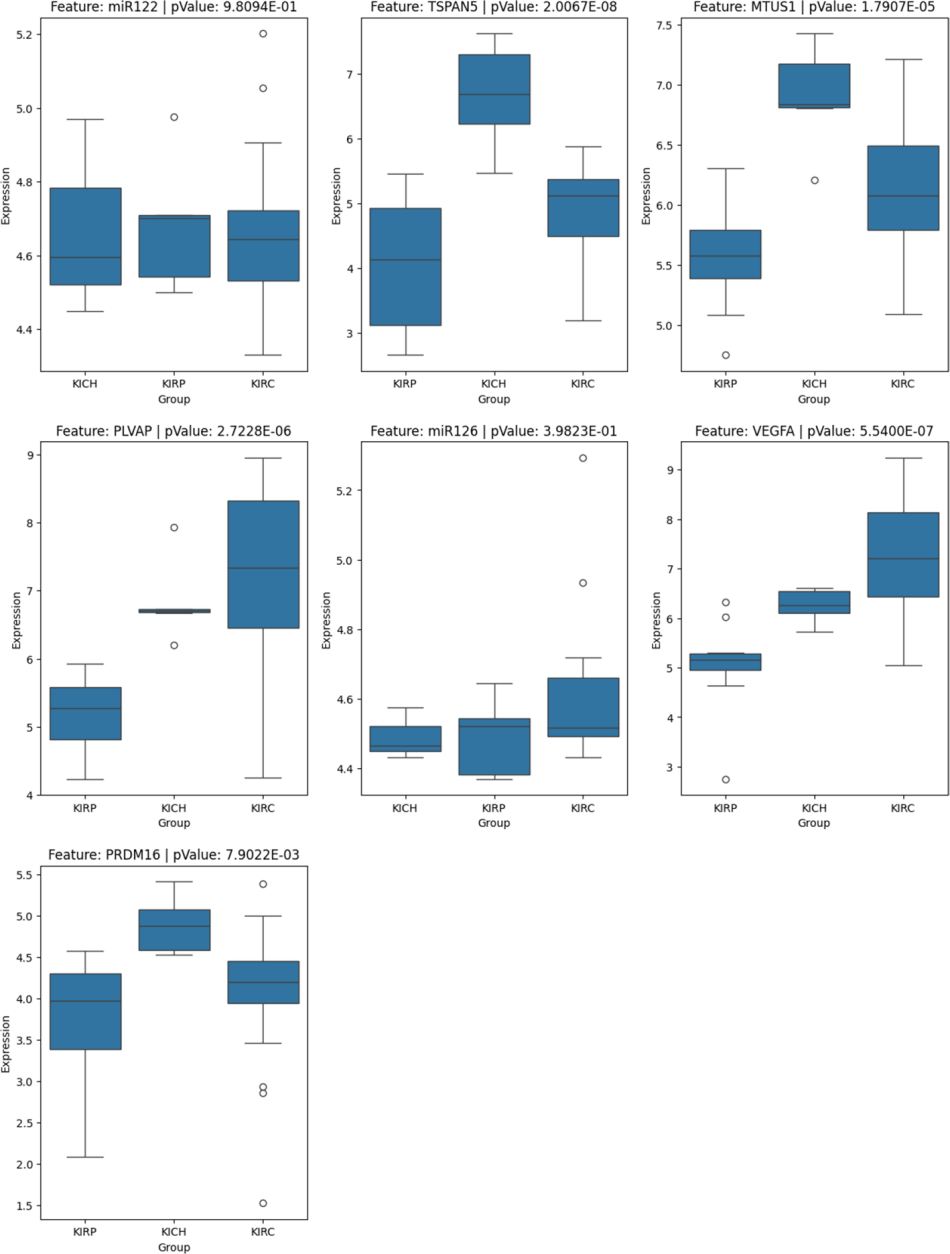
Boxplots showing the expression levels of selected biomarkers across kidney cancer subtypes, with corresponding *p*-values from ANOVA-F tests

Figs. 6 and 7 shows the expression distribution of selected top 10 biomarkers across breast cancer subtypes and kidney cancer subtypes, highlighting their statistical significance based on ANOVA-F test p-values. The results indicate that all selected biomarkers exhibit statistically significant differences across subtypes, with p-values < 0.05 for BRCA-selected biomarkers. These findings further validate the robustness of our biomarker selection and provide strong evidence for their discriminative power in the breast cancer subtype. For KIPAN, the plot confirmed the statistical significance of the majority of the tested biomarkers. However, three biomarkers, SNORD30 (DNA Methy.), SLC22A23 (mRNA), and UBE2N (DNA Methy.) could not be tested due to the limited availability of independent datasets. miR-122 and miR-126 were not found to be statistically significant in the given dataset. Despite these limitations, the overall validation results support the relevance of AMOGEL framework for biomarker identification across different cancer types, particularly BRCA-selected biomarkers.

**Fig. 8:**
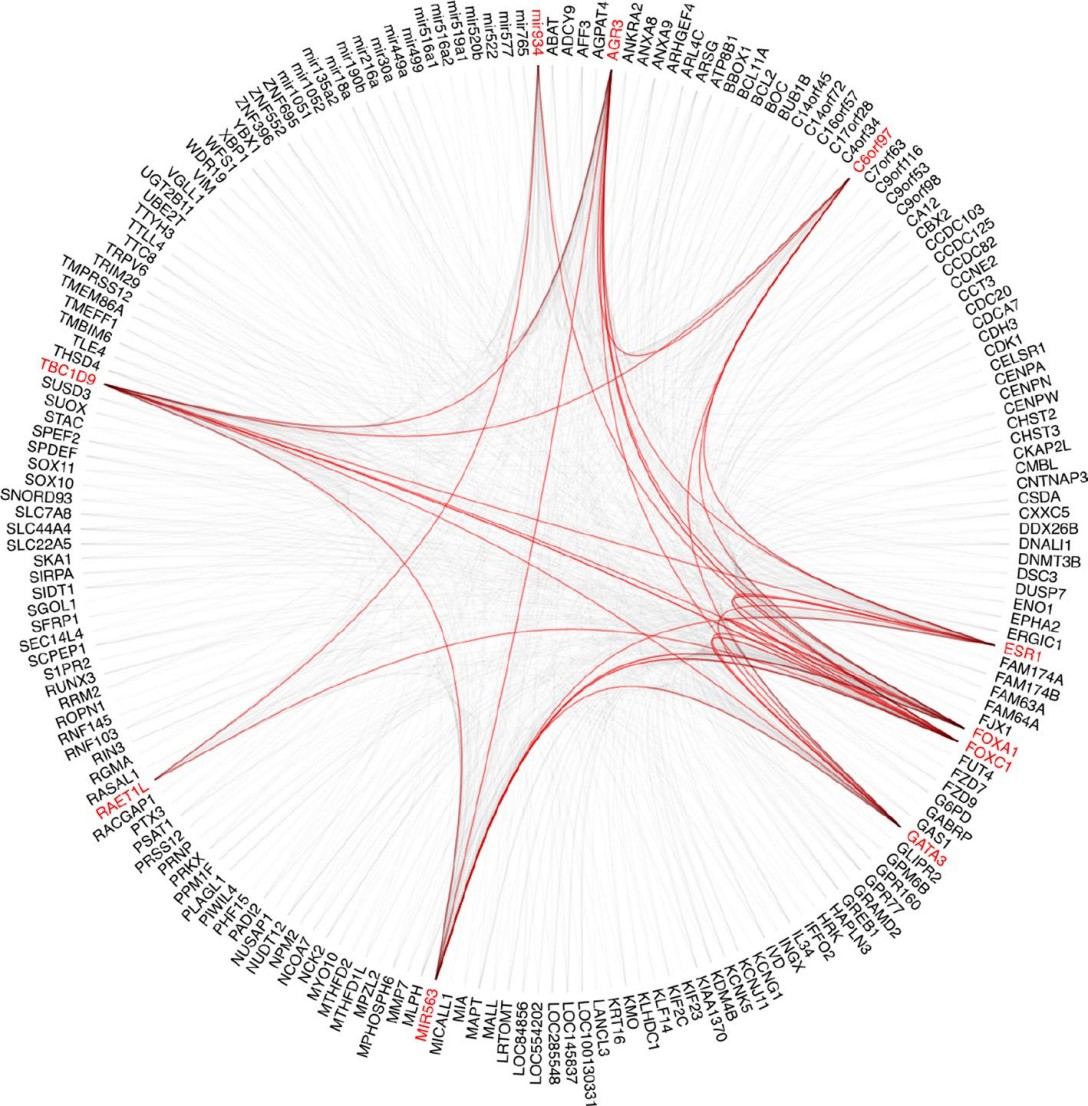
BRCA top 10 genes attention score in relation to top 200 genes. Nodes around the circles represented individual genes and the edges indicated the relationship between the nodes with non-zero attention score. Edges were highlighted in red to show that there are connections (non-zero attention score) within the top 10 genes

**Fig. 9:**
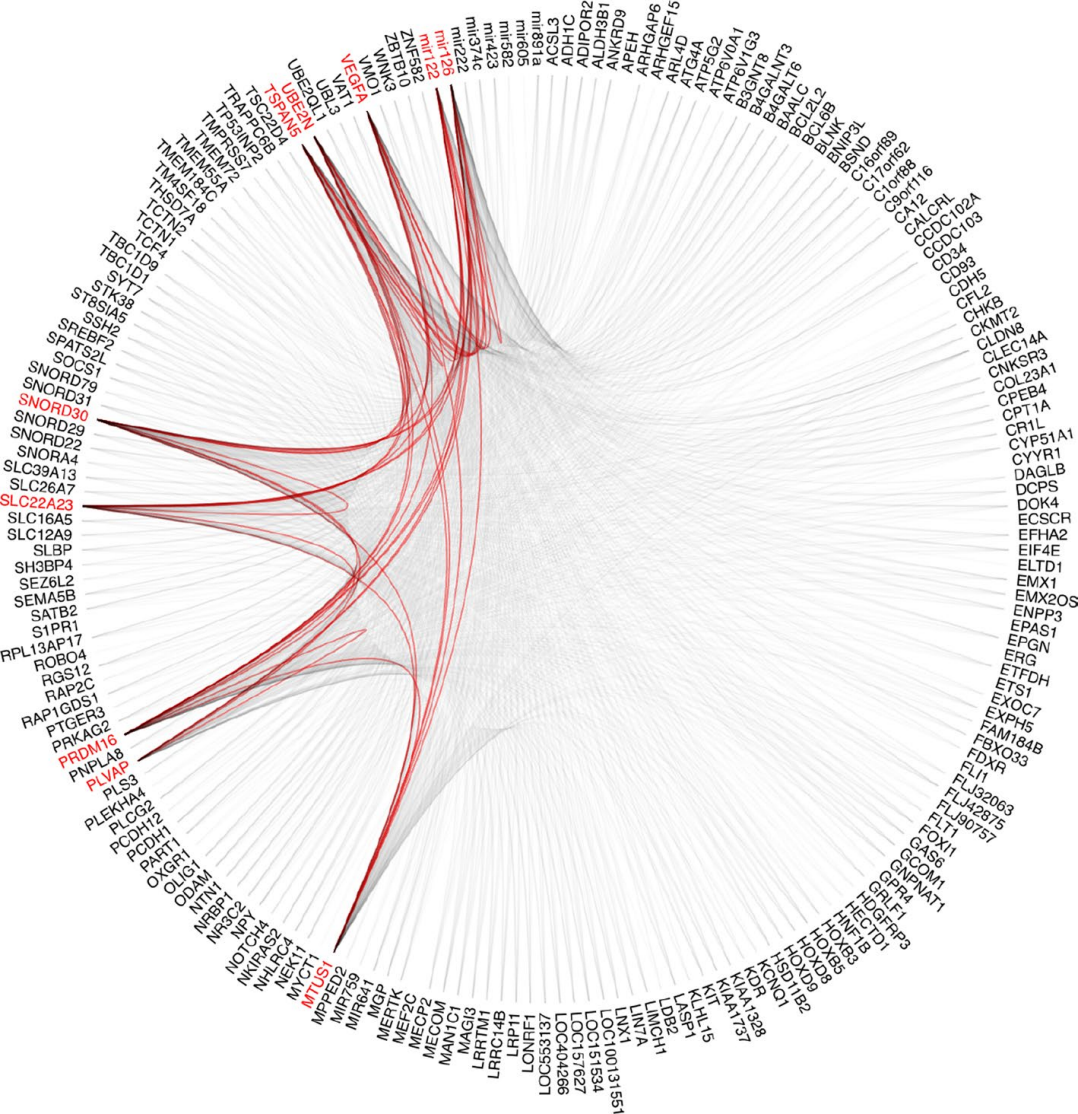
KIPAN top 10 genes attention score in relation to top 200 genes. Nodes around the circles represented individual genes and the edges indicated the relationship between the nodes with non-zero attention score. Edges were highlighted in red to show that there are connections (non-zero attention score) within the top 10 genes

The top 10 genes’ attention scores in relation to the top 200 genes for each dataset were plotted using hierarchical edge bundling, as shown in Figs. 8 and 9. Both the results demonstrated that the top 10 genes are not only significant individually but also share strong mutual interactions, which could be crucial for understanding their collective role in the biological process or condition being studied.

## Conclusion

This study introduced the Associative Multi-Omics Graph Embedding Learning (AMOGEL) model, which effectively integrates multi-omics data and prior biological knowledge through Graph Neural Networks (GNN) and association rule mining (ARM). The model employed an early fusion technique using ARM to mine inter-omics relationships, forming a comprehensive multi-omics graph before model training. By introducing multi-dimensional edges, information edges as the main contributors and prior knowledge edges as auxiliary contributors, AMOGEL enhances the representation of complex biological interactions. Additionally, the gene ranking technique, which utilizes attention scores and the relationships between neighbouring genes, further improves the model’s interpretability and provides useful information on biomarkers discovery.

The integration of miRNA, mRNA, and DNA methylation data with prior knowledge from PPI, KEGG pathways, and Gene Ontology (GO) has shown significant improvement in cancer subtype classification accuracy, F1 score, and AUROC. By generating information-based gene-gene interactions and selecting informative genes, AMOGEL addresses the limitations of existing models that rely on limited prior knowledge. To further validate the ranked biomarkers, we compared the identified top 200 genes with the genetic association database (GAD) from DAVID functional annotation tool. The selected biomarkers exhibited strong enrichment in breast cancer and kidney cancer, with fold enrichment values of up to 4.0 and a false discovery rate (FDR) as low as 1.3%. This enrichment analysis confirms that AMOGEL can effectively rank disease-relevant biomarkers, further reinforcing its biological significance. Additionally, independent validation using external datasets from NCBI GEO confirmed that most selected biomarkers exhibited significant differential expression across subtypes, particularly in BRCA and KIPAN. However, some biomarkers could not be tested due to dataset limitations, and a few were found to be statistically non-significant. Despite the limitations, other promising findings suggest that AMOGEL can serve as a robust framework for enhancing cancer subtype classification and for identifying biologically meaningful biomarkers, which ultimately contribute to more effective and personalized treatment strategies.

The model’s performance is currently influenced by the need for careful hyperparameter tuning, particularly the information-edge filter threshold, to prevent the creation of overly dense graph structures. Future research could focus on developing auto hyperparameter tuning methods and refining ARM algorithms to produce more efficient information-based graphs. While this study focused on the BRCA and KIPAN cancer subtypes, future research should explore the application of AMOGEL to other cancer subtypes to validate its broader applicability.

## Data Availability

Breast cancer subtype, Breast Invasive Carcinoma (BRCA), and kidney cancer subtype, Pan-kidney (KIPAN), dataset can be downloaded from TCGAbiolink (https://bioconductor.org/packages/release/bioc/html/TCGAbiolinks.html) and Broad GDAC Firehouse (https://gdac.broadinstitute.org/). PPI dataset can be downloaded from STRING database https://string-db.org/, while KEGG pathway and GO were retrieved from Database for Annotation, Visualization and Integrated Discovery (DAVID). https://davidbioinformatics.nih.gov/. The source code for AMOGEL can be downloaded from https://github.com/tchiayan/amogel.
